# Supplementary material for: Fungal Immunomodulatory Protein from Nectria haematococca Suppresses Growth of Human Lung Adenocarcinoma by Inhibiting the PI3K/Akt Pathway
Source: Int J Mol Sci. 2018 Nov 1;19(11):3429. doi: 10.3390/ijms19113429 (PMC6274709; doi:10.3390/ijms19113429)
Supplement: Supplementary file 1 [file ijms-19-03429-s001.pdf]

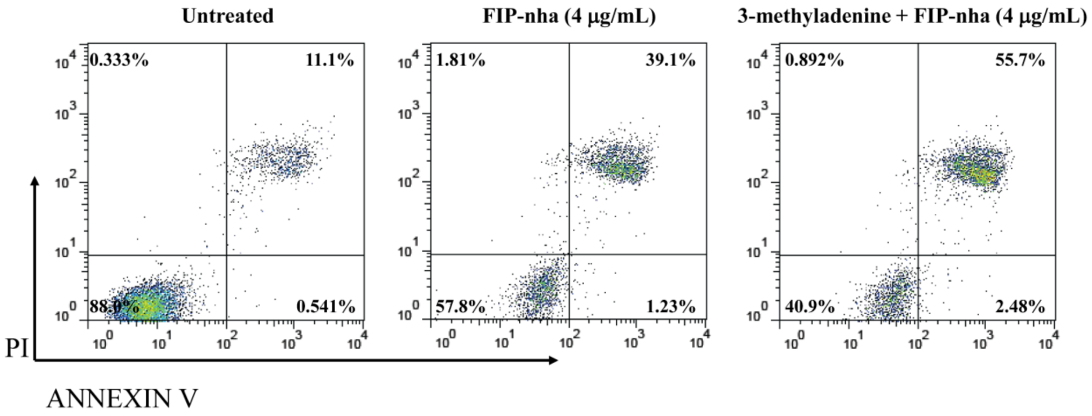

Figure S1. Effects of 3-methyladenine on the apoptosis of A549 cells. The cells were treated with or without FIP-nha (4 μg/mL) and 3-methyladenine (1 mmol) for 24 h. All of the apoptotic levels were labeled using Annexin V-FITC and PI and determined by flow cytometry.
